# Evaluation of optic nerve subarachnoid space in primary open angle glaucoma using ultrasound examination

**DOI:** 10.1371/journal.pone.0208064

**Published:** 2018-11-28

**Authors:** Gilda Cennamo, Daniela Montorio, Maria Angelica Breve, Vincenzo Brescia Morra, Feliciana Menna, Giovanni Cennamo

**Affiliations:** 1 Department of Public Health, University of Naples Federico II, Naples, Italy; 2 Department of Neurosciences, Reproductive Sciences and Dentistry, University of Naples Federico II, Naples, Italy; Faculty of Medicine, Cairo University, EGYPT

## Abstract

**Objectives:**

To measure Optic Nerve Subarachnoid Space (ONSAS) in patients with primary open-angle glaucoma (POAG) and controls using A-scan ultrasound and to evaluate the measurement of the ONSAS in relation to age patient and OCT parameters.

**Methods:**

This retrospective study included 53 consecutive eyes of 27 patients with POAG and 64 normal eyes of 32 controls. Both glaucomatous and control groups were divided into 2 subgroups according to age: <60 age (glaucomatous and control group 1) and 61–90 age (glaucomatous and control group 2).

**Results:**

The ONSAS was significantly lower in all glaucomatous eyes (3.54 ± 0.38) versus normal eyes (3.87 ± 0.32) (p = 0.001). Significant reduction of ONSAS was showed in control group 2 (3.63 mm ± 0.37) compared to control group 1 (3.87 mm ± 0.32) (p = 0.014) and between glaucoma group 1 (3.54 mm ± 0.38) and control group 1 (p = 0.001). While no significant differences were observed between glaucomatous group 2 (3.48 mm ± 0.41) and control group 2 (p = 0.17) and between glaucoma group 1 and glaucoma group 2 (p = 0.609). Lastly, the ONSAS was not significantly associated with GCC and RNFL parameters except for Focal Loss Volume (FLV), Superior RNFL and ONSAS in glaucoma group 1 and for FLV and ONSAS in all glaucomatous group.

**Conclusion:**

Standardized A-scan ultrasound is a non invasive imaging technique with which it is possible to monitor ONSAS changes in glaucomatous patients. The reduction of ONSAS confirm the importance of the lower orbital CSFP as further risk factor in the progression of glaucoma disease.

## Introduction

Primary open angle glaucoma (POAG) is the most common form of glaucoma characterized by a progressive cupping of the optic nerve head due to thinning of the neuroretinal rim, ganglion cell and retinal nerve fiber layers [1].

Increased intraocular pressure (IOP) has long being considered to be the major risk factor for the development and progression of glaucomatous damage [2].

Because many patients, though normal IOP measurements, showed optic nerve damages, additional factors have been hypothesized [3].

The study conducted by Volkov showed that cerebrospinal fluid pressure (CSFP) could pathogenically be correlated with glaucomatous optic neuropathy [4].

In-fact the lamina cribrosa is exposed not only to IOP in the eye but also to CSFP in optic nerve subarachnoid (ONSAS).

The lamina cribrosa separates these two pressurized regions. The pressure drop that occurs across the lamina cribrosa, (IOP-ICP), is known as translaminar pressure difference and is typically directed posteriorly [5].

Different studies have suggested that both elevated IOP and reduced CSFP increase translaminar pressure and play an important role in the pathogenesis and progression of glaucoma disease [6–9].

In previous studies the translaminar pressure difference was evaluated on lumbar CSFP measurement, an invasive technique that reflects the CSFP in ONSAS [10].

This direct measurement of the CSFP is an invasive method and not always acceptable in clinical practice, then it is preferred to use a non-invasive technique to estimate the CSFP in ONSAS.

The purpose of this retrospective study was to measure the ONSAS, using A-scan ultrasound, in POAG and control eyes and to evaluate the ONSAS in relation to age patient and OCT parameters.

## Materials and methods

In this retrospective study we evaluated 53 consecutive eyes of Caucasian 27 patients (16 females, 11 males, mean age 69.8 ± 13.3 years) presenting open-angle glaucoma enrolled from March to June 2016 in the Eye Clinic of the University of Naples “Federico II”. Each patient underwent evaluation of best corrected visual acuity (BCVA) according to the Early Treatment of Diabetic Retinopathy Study (ETDRS), Goldman applanation tonometry, gonioscopy, slit-lamp biomicroscopy, fundus examination with a +90 D lens, standard visual field perimetry (Humprey Field analyzer with Swedish Interactive Thresholding Algorithm SITA standard 30–2 test program (Carl Zeiss Meditec Dublin, CA, USA), RTVue-100 FD-OCT, software version A5, 1, 0, 90 (Optovue, Inc., Fremont, CA, USA) and standardized A-Scan Ultrasound (Aviso S System, Quantel Medical, Bozeman, MT).

Glaucomatous eyes were defined as those with a glaucomatous visual field defect, optic nerve head changes and open angle on gonioscopy. All patients, under topical medication, showed values of IOP within the normal range at the time of ultrasound examination. They were divided according to age: <60 age (glaucomatous group 1: 27 eyes), 61–90 age (glaucomatous group 2: 26 eyes).

The control group was constituted by 32 individuals (18 females, 14 males; for a total of 64 eyes) that had an unremarkable ophthalmic examination, intraocular pressure below 21mmHg, normal visual field tests and OCT examination and no history of glaucoma in a first-degree relative. Also controls were divided according to age: <60 age (control group 1: 32 eyes), 61–90 age (control group 2: 32 eye).

Exclusion criteria for all participants were: current or past ocular pathology that could cause VF changes or optic disc abnormalities; history of intraocular surgery, clinically relevant opacities of the optic media that could influence quality images of OCT; neurological disorder or use of drugs that could influence intracranial pressure; current or past lumbar punctures, cranial surgery and brain trauma.

The study was approved by the Institutional Review Board of the University of Naples “Federico II” and all investigations adhered to the tenets of the Declaration of Helsinki. Written informed consents were obtained from the patients enrolled in the study.

### A-Scan ultrasound

The measurement of the ONSAS was determined by echographic examination using the standardized A-scan method as described by Ossoinig and associates [11–13]. Topical anesthetic was instilled in the conjunctival sac of all participants before starting the exam. After application of gel on the probe, the A-scan probe was placed on the eye in the primary position at lateral canthus and was angled back at the orbital level of the optic nerve until the characteristic optic nerve pattern was obtained on the echographic screen. All measurements were performed at a distance of approximately 2 to 5 mm posterior to the globe. When the sound beam was perpendicular to the surface of the anterior third of the optic nerve, the highest double spikes that corresponded to arachnoid surfaces were obtained from each side of the nerve.

**Fig 1:**
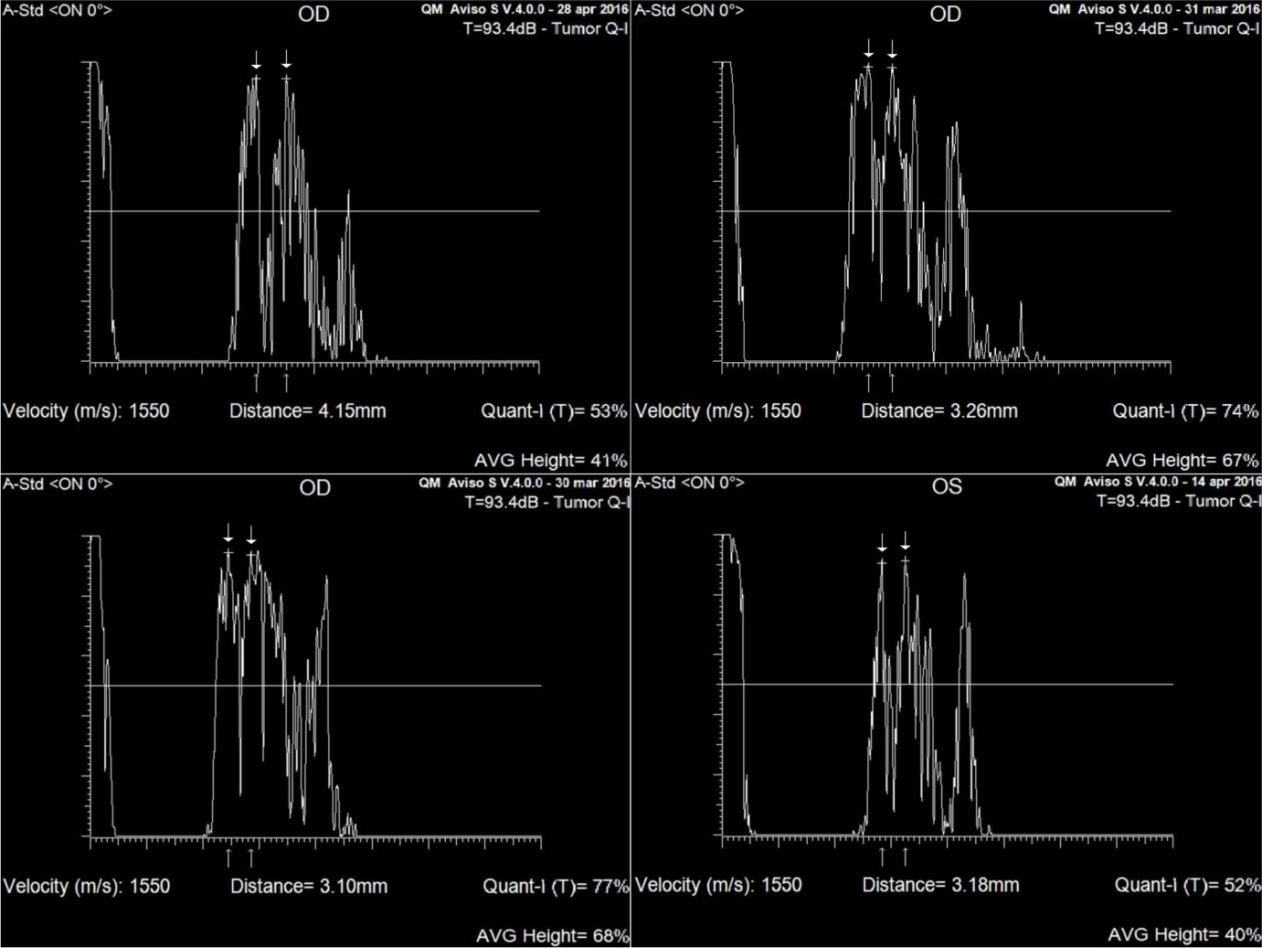
Echograms showing optic nerve subarachnoid space (ONSAS) measurements, using the standardized A-scan ultrasound in control group 1 and 2 in top row (left and right panel, respectively) and in glaucomatous group 1 and 2 in bottom row (left and right panel, respectively). The arrows over the peaks of the two high and perpendicular spikes, as shown in echograms, indicate the maximum arachnoid space diameter measured in the anterior third of the optic nerve.

**Fig 2:**
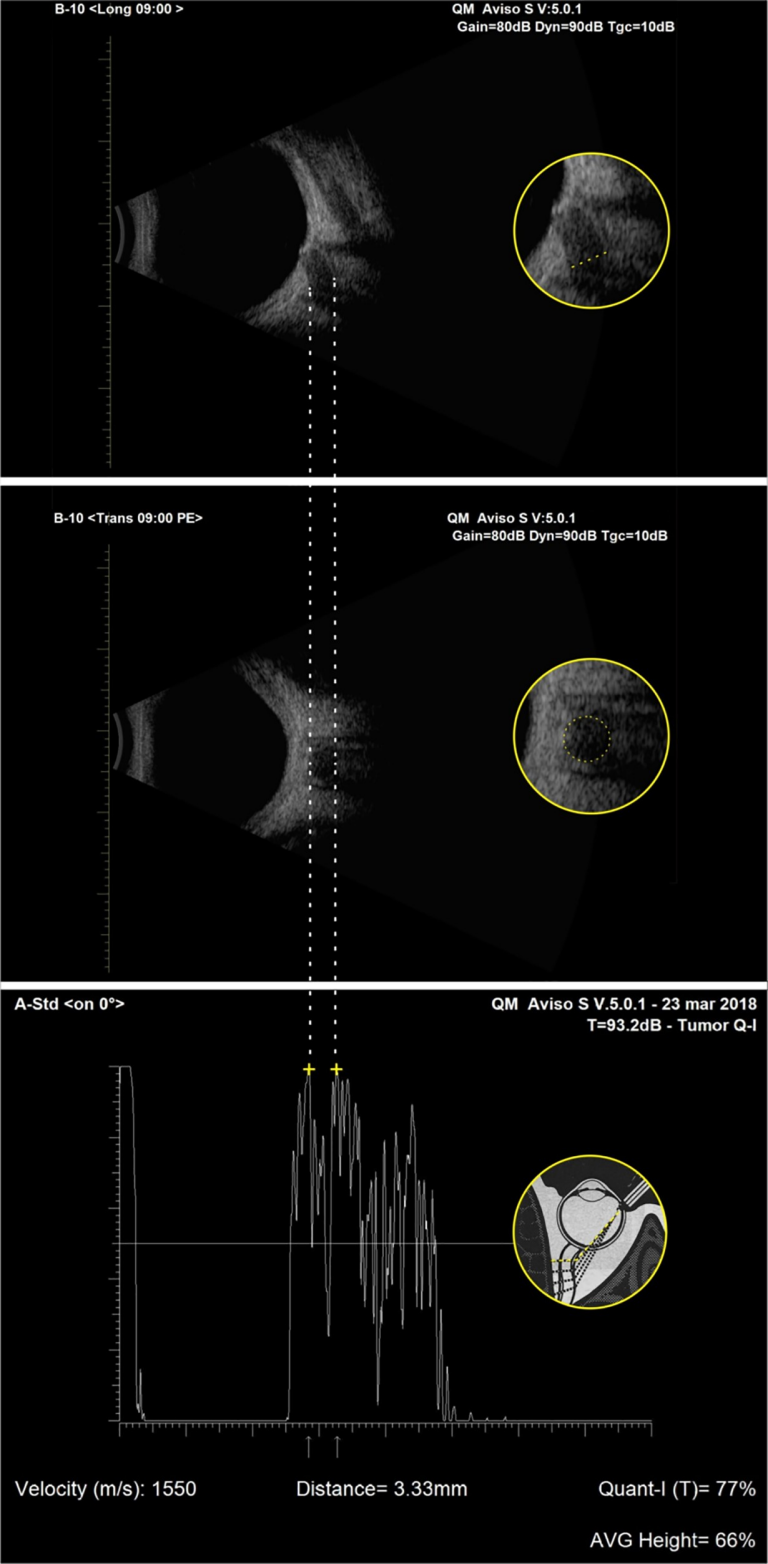
Longitudinal (top) and transverse (middle) B-scan echograms of the optic nerve confirm that the sound beam of A-scan probe is perpendicular to the surface of the anterior third of the optic nerve (yellow dashed line) (bottom).

By placing electronic gates over the peaks of the two maximized arachnoid spikes, the maximum arachnoid space diameter was measured (arrows in Fig 1). Moreover longitudinal and transverse B-scans of the optic nerve were performed to confirm that the sound beam of A-scan probe is perpendicular to the surface of the anterior third of the optic nerve (Fig 2). All measurements were performed by the same experienced examiner not informed with glaucoma diagnosis.

### Spectral domain optical coherence tomography

The retinal nerve fiber layer (RNFL) and ganglion cell complex (GCC) thickness were evaluated, after papillary dilation (minimum diameter 5 mm), with SD-OCT (RTVue-100 OCT, Optovue Inc., Fremont, CA, USA; software version A5, 1, 0, 90) which captures 26,000 axial scans (A-scans) per second and provides a 5-μm depth resolution in tissue. The optic nerve head map protocol was used to evaluate the cpRNFL. This protocol generates a cpRNFL thickness map based on measurements obtained around a circle 3.45 mm in diameter centered on the optic disc. The GCC scan was centered 1-mm temporal to the fovea and covered a square grid (7 × 7 mm) on the central macula, and GCC thickness was measured from the internal limiting membrane to the outer boundary of the inner plexiform layer. Only high-quality images, as defined by a signal strength index above 35, were accepted. The examiner rejected scans that had motion artifacts, poor centration, incorrect segmentation or poor focus [14].

### Statistical analysis

Statistical analysis was performed with the Statistical Package for Social Sciences (Version 20.0 for Windows; SPSS Inc, Chicago, Ill, USA). Student’s t-test analysis for independent samples was used to compare ONSAS values between the glaucomatous and control groups and between patient groups divided according to age. Spearman’s correlation was used to study the associations between ONSAS values and SD-OCT parameters in glaucomatous group. A p value of < 0.05 was considered statistically significant.

## Results

In this retrospective study, 27 patients (16 females, 11 males, mean age 69.8 ± 13.3 years) for a total of 53 eyes were enrolled. Mean BCVA was 0.18 ± 0.2 log MAR and mean IOP was 16.7 ± 2.5 mmHg. The control group was constituted by 32 individuals (18 females, 14 males, mean age 67.7 ± 13.2 years) for a total of 64 eyes examined. There were neither significant age (p = 0.412) or IOP (p = 0.112) differences between patients and controls.

As shown in Table 1, the ONSAS was significantly lower in all glaucomatous eyes (p = 3.54 ± 0.38) versus normal eyes (p = 3.87 ± 0.32) (p = 0.001).

**Table 1:** Demographic and clinical information of glaucomatous and control groups.

	POAG (mean ± SD)	Controls (mean ± SD)	p-value
Eyes, n	53	64	-
Gender (male/female), n	11/16	14/18	-
Age, years	69.8 ± 13.3	67.7 ± 13.2	0.412
IOP (mm hg)	16.7 ± 2.5	16 ± 2.4	0.112
CCT (μm)	552.3 ± 9.3	551.8 ± 8.9	0.769
ONSAS (mm)	3.54 ± 0.38	3.87 ± 0.32	0.001
GCC Average (μm)	91.33 ± 13.73	98.05 ± 4.39	0.001
GCC Superior (μm)	92.59 ± 18.44	99.29 ± 9.21	0.018
GCC Inferior (μm)	90.06 ± 11.44	98.13 ± 4.13	<0.001
FLV (%)	4.01 ± 7.32	1.29 ± 1.12	0.010
GLV (%)	8.46 ± 10.93	3.79 ± 1.82	0.003
RNFL Average (μm)	92.14 ± 15.33	102.68 ± 13.09	<0.001
RNFL Superior (μm)	94.23 ± 17.65	104.45 ± 13.84	0.001
RNFL Inferior (μm)	89.96 ± 15.18	100.97 ± 13.99	<0.001

POAG: Primary Open Angle Glaucoma; IOP: Intraocular Pressure, CCT: Central Corneal thickness

ONSAS: Optical Nerve Subarachnoid Space; GCC: Ganglion Cell Complex; RNFL: Retinal Nerve Fiber Layer.

Data are expressed as mean ± SD except for eyes and gender; Statistical significance P <0.05

**Fig 3:**
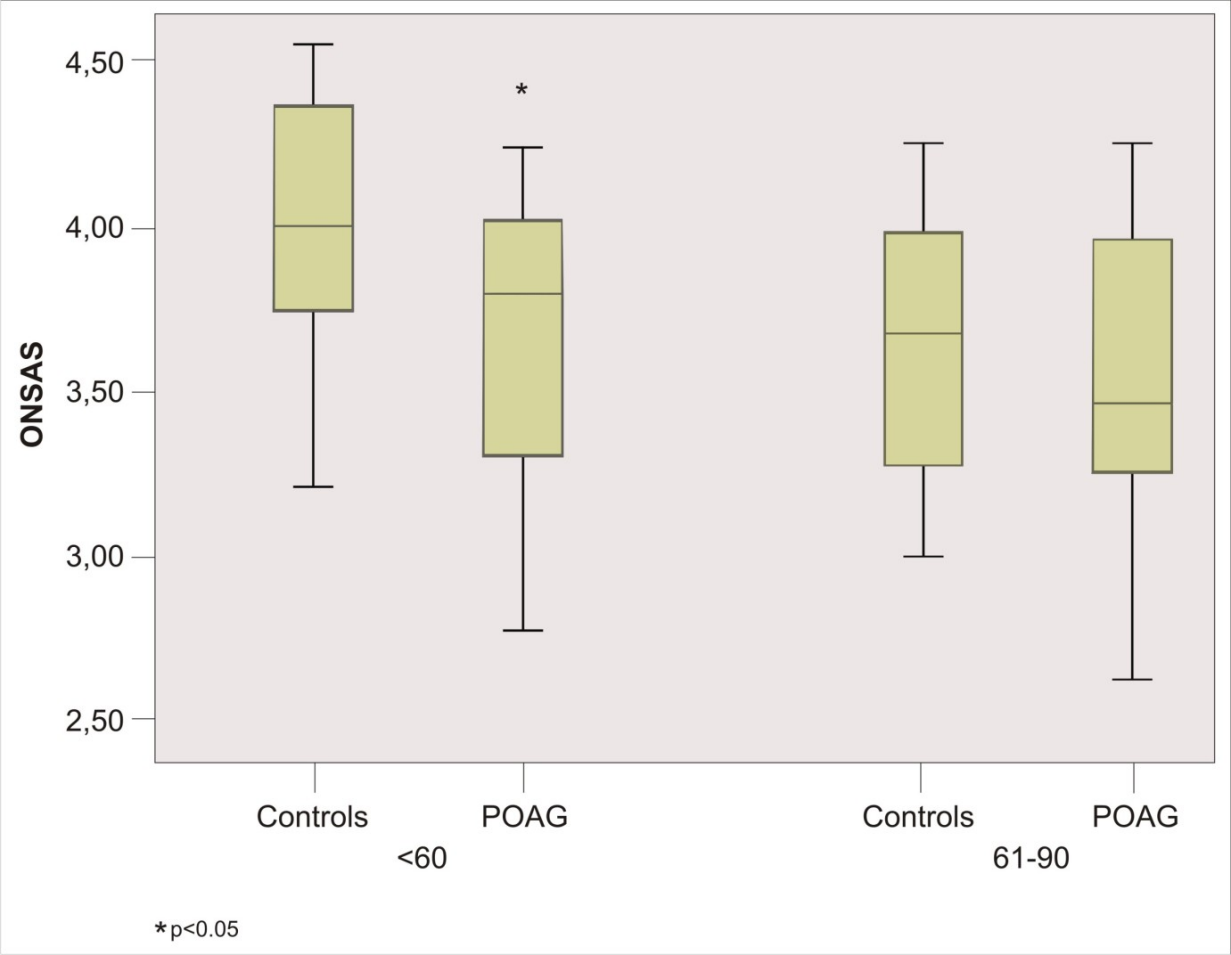
Box plots showing A-scan ultrasound measurements of the optic nerve subarachnoid space (ONSAS) in primary open angle glaucoma (POAG) and control groups that were compared according to age patient.

**Fig 4:**
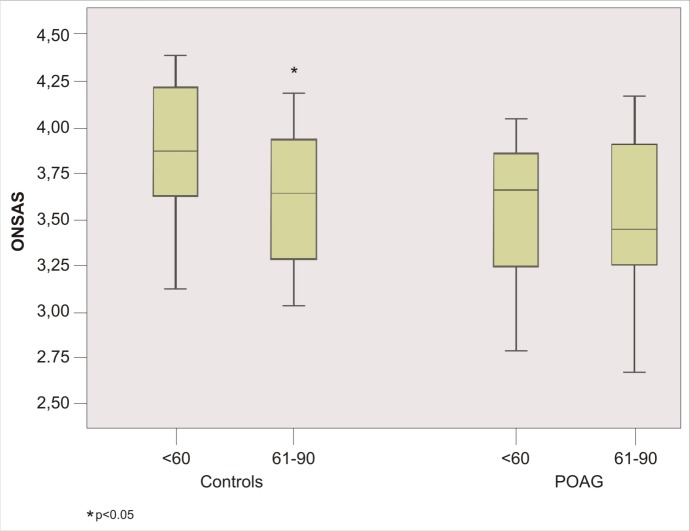
Box plots showing A-scan ultrasound measurements of the optic nerve subarachnoid space (ONSAS) that were compared between the younger (< 60 age) and older (61–90 age) subjects in each glaucomatous and control group.

**Fig 5:**
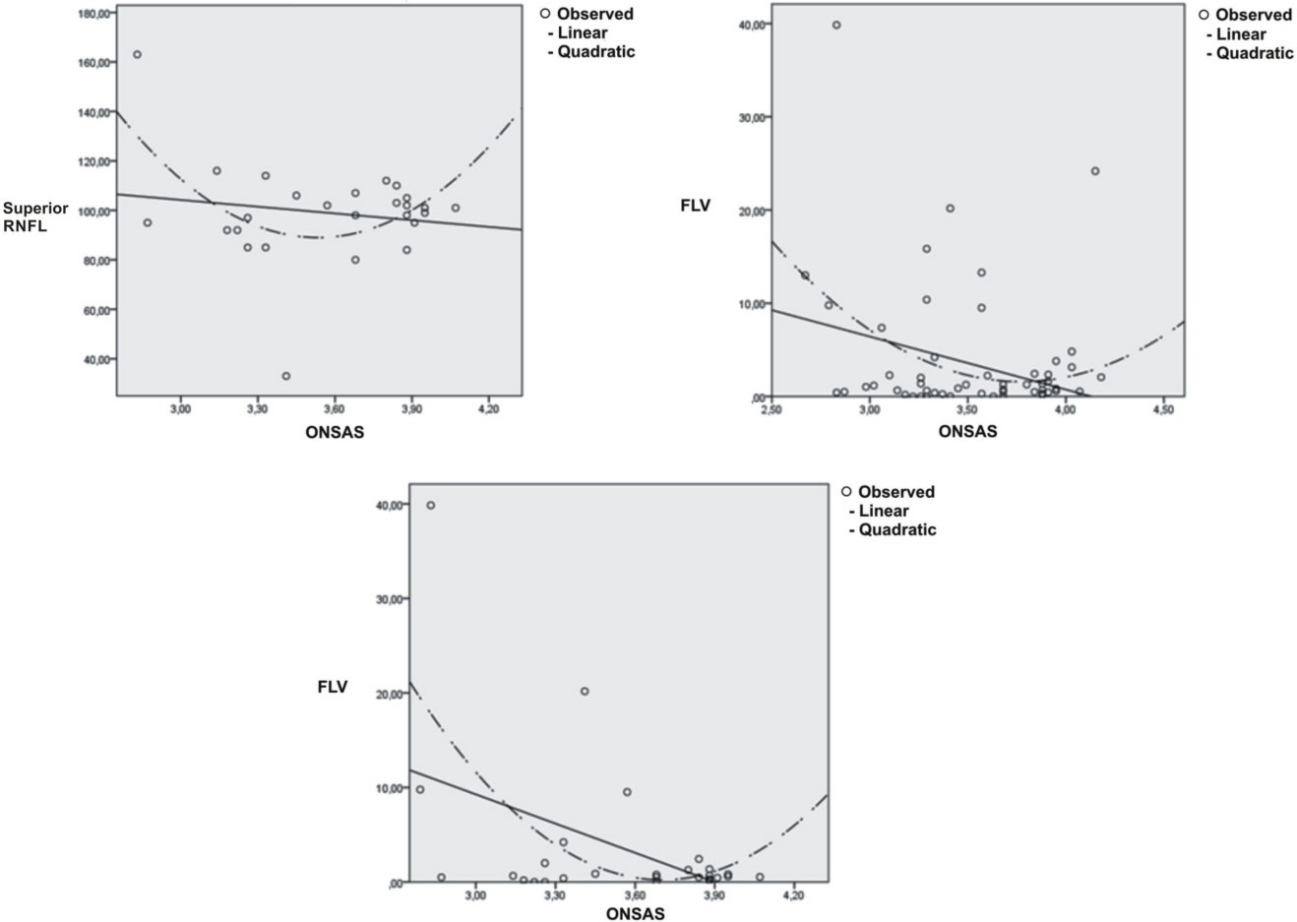
Scatter plots showing significant correlations between the optic nerve subarachnoid space (ONSAS) and superior retinal nerve fiber layer (Superior RNFL) (top left) and between ONSAS and focal loss volume (FLV) in glaucoma group 1 (top right) and between ONSAS and FLV in all glaucomatous group (bottom).

Different results are showed comparing patients and controls divided according to age. ONSAS was significantly different (p = 0.001) between glaucoma group 1 (3.54 mm ± 0.38) and control group 1 (3.87 mm ± 0.32). While ONSAS did not differ (p = 0.17) between glaucomatous groups 2 (3.48 mm ± 0.41) and control group 2 (3.63 mm ± 0.37) (Fig 3). No significant differences were observed between glaucoma group 1 and glaucoma group 2 (p = 0.609). While significant reduction of the ONSAS was showed in control group 2 compared with control group 1 (p = 0.014) (Fig 4). Lastly, no significant correlation was found between OCT parameters and ONSAS of glaucomatous patients, except for Focal Loss Volume (FLV) (P = 0.023), Superior RNFL (p = 0.044) and ONSAS in glaucoma group 1 and for FLV (p = 0.028) and ONSAS in all glaucomatous group (Fig 5).

## Discussion

To our knowledge, this is the first study to use standardized A-Scan ultrasound to evaluate the measurement of ONSAS in POAG patients.

We found a statistically significant reduction in ONSAS in all glaucomatous eyes compared with normal eyes.

Our results are in line with the study conducted by Liu et al. that identified, using B-Scan ultrasound, a reduction of ONSAS in Normal Tension Glaucoma (NTG) and POAG patients respect to controls [15].

In our study we also evaluated the measurement of ONSAS in relation to age patient.

We showed a statistically significant reduction in ONSAS in glaucoma group 1 (< 60 years) compared with control group 1 (< 60 years).

This finding is in line with the study, conducted by Berdhal et al, that showed significant differences in CSFP between POAG group and controls in < 60 age category of patients [7].

The glaucoma contributes to decline of CSFP determining a significant reduction of ONSAS although the progressive reduction of CSFP begins physiologically after age 60, as demonstrated by several studies [9,16].

The comparison between control group 1 (< 60 years) and control group 2 (> 60 years) was also statistically significant confirming different studies that have reported the steady decline of CSFP with a percent reduction from 2.5% for the 50–54 age group to 26.9% for the 90–95 age group in normal subjects [16].

In our study there was no significant differences in ONSAS comparing younger and older glaucomatous patients. The glaucoma have determined the reduction of CSFP in the same way regardless of age patient as demonstrated also by Berdhal et al [7].

Lastly, POAG patients and controls in > 60 age category were characterized by similar reduction of ONSAS.

This result confirms that advanced age and glaucoma disease determine a reduction of CSFP.

The study, conducted by Fleischman et al, found a significant reduction of CSFP after 60 age in normal subjects [17].

Different studies have showed that the age where CSFP started to decline coincided with the age where the prevalence of POAG increased.

The POAG prevalence begins in the 5^th^ and 6^th^ decades and progressively increases through the 8^th^ and 9^th^ decades [18–20].

These data support the hypothesis that reduced CSFP represent a risk factor for progression of POAG and provide an explanation for the mechanism that underlies the age-related increase in the prevalence of POAG.

It has been hypothesized that the choroid plexus that produce CSF within cerebral ventricles undergoes to aging changes determining a reduction of CSF [21–23].

Moreover in plasma of older subjects the levels of vasopressin, a hormone that decrease blood flow of choroid plexus, are increased causing a reduction of CSF production [24,25].

Our study have showed no significant correlation between ONSAS and OCT parameters except between ONSAS and RNFL thickness as confirmed by different studies [15,26].

The changes of GCC and RNFL parameters are the result of a multifactorial pathogenesis that involves not only the reduction of orbital CSFP but also other risk factors such as the ocular blood flow decreased, diabetes, myopia, ethnicity, and genetic background [27–31].

Several studies, as those conducted by Bauerle and Steinborn, reported a good correlation between ultrasound and MRI in measurements of the optic nerve sheath diameter (ONSD) [32,33].

Also Picher et al, revealed no significant difference between the measurements of ONSD in NTG patients performed with CT and those with MRI [34].

Wang et al, using MRI, found that the ONSASW was significantly narrower in NTG than in POAG and control groups at 3, 9 and 15 mm behind the globe. While POAG and control groups did not vary significantly at any of the 3 measuments [35].

While Jaggi et al., using CT scan, showed an increased ONSD in NTG patients compared with controls in prone position [36].

It has been demonstrated that the prone position determines an increased central pressure venous influencing the CSF distribution in the central nervous system [37].

In a recent study conducted by Liu et al., the ONSASW was evaluated by B-scan ultrasound at 3, 5 and 7 mm behind the globe. The ONSASW was significantly narrower in NTG than in the POAG and controls groups, while there no significant differences between the POAG and control groups [15].

Conversely, Pinto et al. have showed, using B-scan ultrasound, that ONSD was not significantly different between control, NTG and POAG patients [26].

MRI and CT are non-invasive techniques with high spatial resolution but the high costs restrict their wider use as follow-up tools.

The ultrasound assessment of ONSAS is easily available method and cost-effective. It is a non-invasive technique characterized by a robust scan-rescan reproducibility [32].

The evaluation of ONSAS in glaucomatous patients has until recently possible by B-scan ultrasound that does not always performs standard measurements [38,39].

In fact the studies conducted by Liu and Pinto, using B-scan ultrasound have reported different results and a possible explanation could be given by different probe used.

Liu et al. employed a 12 MHz ultrasound probe with a resolution of the ultrasound images higher than the 7.5 MHz probe used in the study of Pinto et al. that may have influenced the accuracy of measurements [15,26].

Moreover the transbulbar sound direction and the incidence of the ultrasound beam on the lamina cribrosa or the dura madre may produce acoustic shadows behind the globe influencing precise measurements [15,40].

Accurate method that provides reliable images of ONSAS measuring can be obtained by the standardized A-scan ultrasound.

It is a simple, sensitive and non-invasive method of detecting and characterizing of ONSAS and allows a real-time examination [41–43].

It is important that sound beam is directed in a perpendicular direction toward the surface of the optic nerve.

When the sound beam incidence is in this position, A-scan ultrasound echograms showed high and perperdicular double spikes that correspond to arachnoidal surfaces [11–13].

The distance between the these two steeply rising surface spikes indicates the maximum arachnoidal diameter in the area scanned by the beam.

To analyze the anterior third of the optic nerve in the point in which are obtained these high and perperdicular double spikes allows to acquire reproducible and repeatable measurements of ONSAS [11,12].

Until now the A-scan ultrasound was used to measure the maximal pial diameter in POAG patients showing a significant reduction compared to controls [44].

Whereas in this study the measurement of ONSAS, using the same tool, have showed a valid and indirect method of CSFP evaluation.

Different studies have showed, using various measurement methods, the close correlation between intracranial pressure (ICP) and ONSD: even slight variations of ICP influences the diameter of the optic nerve [45–51].

In fact, unlike the brain, the optic nerve is not surrounded by a rigid structure, but by orbital fat which is easily compressible.

Thus, any increasing pressure variations of CSF provokes swelling of the optic nerve sheaths owing to the increase in the quantity of internal fluid which subsequently increases the diameter of the optic nerve.

Already in the 1987, Cangemi et al, have showed that the measurement of ONSD by means of A-scan ultrasound provided an immediate evaluation of ICP.

Any increased of CSFP in patients with intracranial hypertension reflected in real time the dilated ONSD also after the surgical treatment when ONSD returned to the normal size showing an immediate reduction of CSFP [52].

The limitation of this study is the relatively small sample size of the groups of patients. Future studies on larger cohorts for each study group are needed to further evaluate the changes of ONSAS in POAG patients.

In conclusion, standardized A-scan ultrasound represents a non-invasive imaging technique with which it is possible to monitor ONSAS changes in glaucomatous patients. The reduction of ONSAS, measured by this tool, confirms the involvement of the lower orbital CSFP in the translaminar pressure gradient that plays an important role in the pathogenesis and progression of POAG.

## Supporting information

S1 Table Measurements of the optic nerve subarachnoid space in glaucoma groups 1 and 2. (PDF)

## References

[1] Hitchings RA, Spaeth GL. The optic disc in glaucoma. I: classification. Br J Ophthalmol. 1976; 60:778–785 1009058 10.1136/bjo.60.11.778 PMC1042838

[2] Leske MC, Heijl A, Hussein M, Bengtsson B, Hyman L, Komaroff E, et al Factors for glaucoma progression and the effect of treatment: the Early Manifest Glaucoma Trial. Arch Ophthalmol. 2003; 121:48–56. 12523884 10.1001/archopht.121.1.48

[3] Drance SM. Some factors in the production of low tension glaucoma. Br J Ophthalmol. 1972; 56:229–242 5032759 10.1136/bjo.56.3.229 PMC1208757

[4] Volkov VV. Essential element of the glaucomatous process neglected in clinical practice. Oftalmol Zh. 1976; 31: 500–504. 1012622

[5] Morgan WH, Yu DY, Alder VA, Cringle SJ, Cooper RL, House PH, et al The correlation between cerebrospinal fluid pressure and retrolaminar tissue pressure. Invest Ophthalmol Vis Sci 1998; 39:1419–1428. 9660490

[6] Jonas JB, Berenshtein E, Holbach L. Anatomic relationship between lamina cribrosa, intraocular space, and cerebrospinal fluid space. Invest Ophthalmol Vis Sci 2003; 44: 5189–5195. 14638716 10.1167/iovs.03-0174

[7] Berdahl JP, Allingham R, Johnson DH. Cerebrospinal fluid pressure is decreased in primary open-angle glaucoma. Ophthalmology. 2008; 115: 763–768. 10.1016/j.ophtha.2008.01.013 18452762 10.1016/j.ophtha.2008.01.013

[8] Berdhal JP, Fautsch MP, Stinnett SS, Allingham RR. Intracranial pressure in primary open angle glaucoma, normal tension glaucoma, and ocular hypertension: a casa-control study. Invest Ophthalmol Vis Sci. 2008; 49: 5412–5418. 10.1167/iovs.08-2228 18719086 10.1167/iovs.08-2228 PMC2745832

[9] Ren R, Zhang X, Wang N, Li B, Tian G, Jonas JB. Cerebrospinal fluid pressure in ocular hypertension. Acta Ophthalmol 2011; 89: e142–148. 10.1111/j.1755-3768.2010.02015.x 21348961 10.1111/j.1755-3768.2010.02015.x

[10] Hou R, Zhang Z, Yang D, Wang H, Chen W, Li Z, et al Intracranial pressure (ICP) and optic nerve subarachnoid space pressure (ONSP) correlation in the optic nerve chamber: the Beijing Intracranial and Intraocular Pressure (iCOP) study. Brain Res 2016; 1635: 201–208. 10.1016/j.brainres.2016.01.011 26794252 10.1016/j.brainres.2016.01.011

[11] Ossoining KC. Standardized echography of the optic nerve In: Till P, eds. Documenta Ophthalmologica Proceedings Series vol 55. Ophthalmic echography 13, Dordrecht: Springer Netherlands, 1990: 3–99

[12] Ossoining KC. Standardized Echography: basic principles, clinical applications and results. Ophthalmic ultrasonography: comparative technique Dallow R. L. (ed.) Int. Ophthal. Clin. 19/4, Little Brown Co, Boston, 1979 395120

[13] Ossoining KC, Cennamo G, Frazier-Byrne S. Echographic differential diagnosis of optic nerve lesions In: Thijssen JM, Verbek AM, eds, Documenta Ophthalmologica Proceedings Series vol 29, Ultrasonography in Ophthalmology, The Hague: Springer Netherlands: 1981:327–332.

[14] Hirashima T, Hangai M, Nukada M, Nakano N, Morooka S, Akagi T, et al Frequency-doubling technology and retinal measurements with spectral-domain optical coherence tomography in preperimetric glaucoma. Graefes Arch Clin Exp Ophthalmol. 2013;251:129–137. 10.1007/s00417-012-2076-7 22684903 10.1007/s00417-012-2076-7

[15] Liu H, Yang D, Ma T, Shi W, Zhu Q, Kang J, et al Measurement and Associations of the Optic Nerve Subarachnoid Space in Normal Tension and Primary Open-Angle Glaucoma. Am J Ophthalmol. 2018;186:128–137. 10.1016/j.ajo.2017.11.024 29246580 10.1016/j.ajo.2017.11.024

[16] Fleischman D, Allingham RR. The role of cerebrospinal fluid pressure in glaucoma and other ophthalmic diseases: A review. Saudi J Ophthalmol 2013;27:97–106. 10.1016/j.sjopt.2013.03.002 24227969 10.1016/j.sjopt.2013.03.002 PMC3809480

[17] Fleischman D, Berdahl JP, Zaydlarova J, Stinnett S, Fautsch MP, Allingham RR. Cerebrospinal fluid pressure decreases with older age. PLoS One. 2012;7:e52664 10.1371/journal.pone.0052664 23300737 10.1371/journal.pone.0052664 PMC3530461

[18] Friedman DS, Wolfs RC, O'Colmain BJ, Klein BE, Taylor HR, West S, et al Prevalence of open-angle glaucoma among adults in the United States. Arch Ophthalmol. 2004;122:532–538. 10.1001/archopht.122.4.532 15078671 10.1001/archopht.122.4.532 PMC2798086

[19] Quigley HA, Broman AT. The number of people with glaucoma worldwide in 2010 and 2020. Br J Ophthalmol. 2006; 90: 262–267. 10.1136/bjo.2005.081224 16488940 10.1136/bjo.2005.081224 PMC1856963

[20] Yoshida M, Okada E, Mizuki N, Kokaze A, Sekine Y, Onari K, et al Age-specific prevalence of open-angle glaucoma and its relationship to refraction among more than 60,000 asymptomatic Japanese subjects. J Clin Epidemiol. 2001;54: 1151–1158. 11675167 10.1016/s0895-4356(01)00388-2

[21] May C, Kaye JA, Atack JR, Schapiro MB, Friedland RP, Rapoport SI, et al Cerebrospinal fluid production is reduced in healthy aging. Neurology. 1990;40 (3 Pt 1): 500–503. 2314595 10.1212/wnl.40.3_part_1.500

[22] Redzic ZB, Preston JE, Duncan JA, Chodobski A, Szmydynger-Chodobska J. The choroid plexus cerebrospinal fluid system: from development to aging. Curr Top Dev Biol. 2005; 71: 1–52. 10.1016/S0070-2153(05)71001-2 16344101 10.1016/S0070-2153(05)71001-2

[23] Serot JM, Bene MC. Faure GC. Choroid plexus, aging of the brain, and Alzheimer’s disease. Front Biosci. 2006; 8: s515–521. 10.2741/1085 12700093

[24] Faraci FM, Mayhan WG, Heisted DD. Effect of vasopressin on cerebrospinal fluid production: possible role of vasopressin V1 receptors. Am J Physiol. 1990; 258(1 Pt 2): R94–99 10.1152/ajpregu.1990.258.1.R94 2137302 10.1152/ajpregu.1990.258.1.R94

[25] Frolkis VV, Kvitnitskaia-Ryzhova T, Dubiley TA. Vasopressin, hypothalmol-neurohypophyseal system and aging. Arch Geront Ger. 1990; 29: 109–214. 10.1016/s0167-4943(99)00032-1 15374053

[26] Abegão Pinto L, Vandewalle E, Pronk A, Stalmans I. Intraocular pressure correlates with optic nerve sheath diameter in patients with normal tension glaucoma. Graefes Arch Clin Exp Ophthalmol. 2012;250:1075–80. 10.1007/s00417-011-1878-3 22160505 10.1007/s00417-011-1878-3

[27] Gherghel D, Hosking SL, Orgul S. Autonomic nervous system, circadian rhythms, and primary open-angle glaucoma. Surv Ophthalmol. 2004;49:491–508. 10.1016/j.survophthal.2004.06.003 15325194 10.1016/j.survophthal.2004.06.003

[28] Grieshaber MC, Flammer J. Blood flow in glaucoma. Curr Opin Ophthalmol. 2005; 16:79–83. 15744136 10.1097/01.icu.0000156134.38495.0b

[29] Nakamura M, Kanamori A, Negi A. Diabetes mellitus as a risk factor for glaucomatous optic neuropathy. Ophthalmologica. 2005; 219:1–10. 10.1159/000081775 15627820 10.1159/000081775

[30] Xu L, Wang Y, Wang S, Wang Y, Jonas JB. High myopia and glaucoma susceptibility; the Beijing Eye Study. Ophthalmology. 2007;114:216–220. 10.1016/j.ophtha.2006.06.050 17123613 10.1016/j.ophtha.2006.06.050

[31] Wolfs RC, Klaver CC, Ramrattan RS, van Duijn CM, Hofman A, de Jong PT. Genetic risk of primary open-angle glaucoma. Population-based familial aggregation study. Arch Ophthalmol. 1998;116:1640–1645. 9869795 10.1001/archopht.116.12.1640

[32] Bäuerle J, Schuchardt F, Schroeder L, Egger K, Weigel M, Harloff A. Reproducibility and accuracy of optic nerve sheath diameter assessment using ultrasound compared to magnetic resonance imaging. BMC Neurol. 2013;13:187 10.1186/1471-2377-13-187 24289136 10.1186/1471-2377-13-187 PMC4219451

[33] Steinborn M, Fiegler J, Ruedisser K, Hapfelmeier A, Denne C, Macdonald E, et al Measurement of the Optic Nerve Sheath Diameter in Children: Comparison Between Transbulbar Sonography and Magnetic Resonance Imaging. Ultraschall Med. 2011. 10.1055/s-0031-1273491 21870318

[34] Pircher A, Montali M, Berberat J, Remonda L, Killer HE. Relationship between the optic nerve sheath diameter and lumbar cerebrospinal fluid pressure in patients with normal tension glaucoma. Eye (Lond). 2017;31:1365–1372 28452990 10.1038/eye.2017.70 PMC5601451

[35] Wang N, Xie X, Yang D, Xian J, Li Y, Ren R, et al Orbital cerebrospinal fluid space in glaucoma: the Beijing intracranial and intraocular pressure (iCOP) study. Ophthalmology. 2012;119:2065–2073. 10.1016/j.ophtha.2012.03.054 22749084 10.1016/j.ophtha.2012.03.054

[36] Jaggi GP, Miller NR, Flammer J, Weinreb RN, Remonda L, Killer HE. Optic nerve sheath diameter in normal-tension glaucoma patients. Br J Ophthalmol. 2012;96:53–56. 10.1136/bjo.2010.199224 21398413 10.1136/bjo.2010.199224

[37] Schwartz KM, Luetmer PH, Hunt CH, Kotsenas AL, Diehn FE, Eckel LJ, et al Position-related variability of CSF opening pressure measurements. AJNR Am J Neuroradiol. 2013;34:904–907. 10.3174/ajnr.A3313 23064593 10.3174/ajnr.A3313 PMC7964481

[38] De Bernardo M, Rosa N. Clarification on using ultrasonography to detect intracranial pressure. JAMA Ophthalmol 2017;135:1004–1005. 10.1001/jamaophthalmol.2017.2597 28772288 10.1001/jamaophthalmol.2017.2597

[39] Rosa N, De Bernardo M. Measurement of the optic nerve in a resource-limited setting. J Neurosci Rural Pract 2017; 8:310–311. 10.4103/0976-3147.203830 28479823 10.4103/0976-3147.203830 PMC5402515

[40] Copetti R, Cattarossi L. Optic nerve ultrasound: artifacts and real images. Intensive Care Med. 2009;35:1488–1489; 10.1007/s00134-009-1494-4 19367390 10.1007/s00134-009-1494-4

[41] Rosa N, De Bernardo M. Ultrasound assessment optic nerve sheath diameter in healthy volunteers. J Crit Care 2017; 40:279 10.1016/j.jcrc.2017.03.018 28372887 10.1016/j.jcrc.2017.03.018

[42] De Bernardo M, Marotta G, Rosa N. Sonography of the optic nerve sheath diameter. J Ultrasound Med. 2017 10.1002/jum.14486 29148071

[43] Cennamo G, Rosa N, La Rana A, Pastena B. Standardized A-scan echography and the normal optic nerve. Experience with the new Mini A equipment. Acta Ophthalmol Suppl. 1992; (204): 87–89. 1332404 10.1111/j.1755-3768.1992.tb04934.x

[44] Dichtl A, Jonas JB. Echographic measurement of optic nerve thickness correlated with neuroretinal rim area and visual field defect in glaucoma. Am J Ophthalmol. 1996;122:514–519. 8862048 10.1016/s0002-9394(14)72111-7

[45] Cennamo G, Gangemi M, Stella L. The correlation between endocranial pressure and optic nerve diameter: an ultrasonographic study. In: Ossoining KC (ed) Doc Ophthal Proc Ser 48, 1987 pp. 603–606. Junk Publ

[46] Cennamo G, Sorrentino A, Scannp E, Rosa N. Echographic study of the optic nerve during anesthesia. Orbit, 1985; 4:231–234.

[47] Watanabe A, Kinouchi H, Horikoshi T, Uchida M, Ishigame K. Effect of intracranial pressure on the diameter of the optic nerve sheath. J Neurosurg 2008 109: 255–258. 10.3171/JNS/2008/109/8/0255 18671637 10.3171/JNS/2008/109/8/0255

[48] Newman WD, Hollman AS, Dutton GN, Carachi R. Measurement of optic nerve sheath diameter by ultrasound: a means of detecting acute raised intracranial pressure in hydrocephalus. Br J Ophthalmol 2002; 86: 1109–1113. 12234888 10.1136/bjo.86.10.1109 PMC1771326

[49] Gass A, Barker GJ, Riordan-Eva P, MacManus D, Sanders M, Tofts PS, et al MRI of the optic nerve in benign intracranial hypertension. Neuroradiology 1996; 38:769–773. 8957802 10.1007/s002340050344

[50] Imamura Y, Mashima Y, Oshitari K, Oguchi Y, Momoshima S, Shiga H. Detection of dilated subarachnoid space around the optic nerve in patients with papilloedema using T2 weighted fast spin scho imaging. J Neurol Neurosurg Psychiatry. 1996; 60: 108–109. 8558139 10.1136/jnnp.60.1.108 PMC486205

[51] Wang LJ, Chen LM, Chen Y, Bao LY1, Zheng NN, Wang YZ, et al Ultrasonography Assessments of Optic Nerve Sheath Diameter as a Noninvasive and Dynamic Method of Detecting Changes in Intracranial Pressure. JAMA Ophthalmol. 2018;136:250–256. 10.1001/jamaophthalmol.2017.6560 29392301 10.1001/jamaophthalmol.2017.6560 PMC5885896

[52] Gangemi M, Cennamo G, Maiuri F, D'Andrea F. Echographic measurement of the optic nerve in patients with intracranial hypertension. Neurochirurgia (Stuttg). 1987;30:53–55. 3574583 10.1055/s-2008-1053656

