# Supplementary material for: Evaluation of optic nerve subarachnoid space in primary open angle glaucoma using ultrasound examination
Source: PLoS One. 2018 Nov 28;13(11):e0208064. doi: 10.1371/journal.pone.0208064 (PMC6261615; doi:10.1371/journal.pone.0208064)
Supplement: S1 Table — (PDF) [file pone.0208064.s001.pdf]

S1. Measurements of the Optic Nerve Subarachnoid Space (ONSAS) in Glaucoma groups 1 and 2

Glaucoma Group 1		ONSAS (mm)		Glaucoma Group 2		ONSAS (mm)
Patient 1	Right Eye	3.26		Patient 15	Right Eye	3.26
	Left Eye	2.79			Left Eye	2.83
Patient 2	Right Eye	3.84		Patient 16	Right Eye	3.1
	Left Eye	3.8			Left Eye	3.02
Patient 3	Right Eye	3.33		Patient 17	Right Eye	3.29
	Left Eye	3.14			Left Eye	3.57
Patient 4	Left Eye	2.83		Patient 18	Right Eye	3.95
Patient 5	Right Eye	3.88			Left Eye	3.06
	Left Eye	4.07		Patient 19	Right Eye	3.41
Patient 6	Right Eye	3.26			Left Eye	3.29
	Left Eye	3.33		Patient 20	Right Eye	3.37
Patient 7	Right Eye	3.22			Left Eye	3.63
	Left Eye	3.18		Patient 21	Right Eye	4.03
Patient 8	Right Eye	2.87			Left Eye	4.18
	Left Eye	3.45		Patient 22	Right Eye	3.29
Patient 9	Right Eye	3.57			Left Eye	2.67
	Left Eye	3.41		Patient 23	Right Eye	3.29
Patient 10	Right Eye	3.68			Left Eye	3.57
	Left Eye	3.88		Patient 24	Right Eye	3.68
Patient 11	Right Eye	3.68			Left Eye	3.91
	Left Eye	3.95		Patient 25	Right Eye	3.49
Patient 12	Right Eye	3.68			Left Eye	2.98
	Left Eye	3.88		Patient 26	Right Eye	4.15
Patient 13	Right Eye	3.91			Left Eye	4.03
	Left Eye	3.84		Patient 27	Right Eye	3.91
Patient 14	Right Eye	3.88			Left Eye	3.6
	Left Eye	3.95				
